# The Regulatory NOD-Like Receptor NLRC5 Promotes Ganglion Cell Death in Ischemic Retinopathy by Inducing Microglial Pyroptosis

**DOI:** 10.3389/fcell.2021.669696

**Published:** 2021-05-20

**Authors:** Yang Deng, Yunzhao Fu, Longxiang Sheng, Yixin Hu, Lishi Su, Jiawen Luo, Chun Yan, Wei Chi

**Affiliations:** State Key Laboratory of Ophthalmology, Zhongshan Ophthalmic Center, Sun Yat-sen University, Guangzhou, China

**Keywords:** NLRC5, retinal ischemia–reperfusion injury, pyroptosis, apoptosis, microglia

## Abstract

Retinal ischemia is a common pathological event that can result in retinal ganglion cell (RGC) death and irreversible vision loss. The pathogenic mechanisms linking retinal ischemia to RGC loss and visual deficits are uncertain, which has greatly hampered the development of effective treatments. It is increasingly recognized that pyroptosis of microglia contributes to the indirect inflammatory death of RGCs. In this study, we report a regulatory NOD-like receptor, NOD-, LRR- and CARD-containing 5 (NLRC5), as a key regulator on microglial pyroptosis and the retinal ischemia process. Through an in-depth analysis of our recently published transcriptome data, we found that NLRC5 was significantly up-regulated in retina during ischemia–reperfusion injury, which were further confirmed by subsequent detection of mRNA and protein level. We further found that NLRC5 was upregulated in retinal microglia during ischemia, while NLRC5 knockdown significantly ameliorated retinal ischemic damage and RGC death. Mechanistically, we revealed that knockdown of NLRC5 markedly suppressed gasdermin D (GSDMD) cleavage and activation of interleukin-1β (IL-1β) and caspase-3, indicating that NLRC5 promotes both microglial pyroptosis and apoptosis. Notably, we found that NLRC5 directly bound to NLRP3 and NLRC4 in inflammasomes to cooperatively drive microglial pyroptosis and apoptosis mediating retinal ischemic damage. Overall, these findings reveal a previously unidentified key contribution of NLRC5 signaling to microglial pyroptosis under ischemia or hypoxia conditions. This NLRC5-dependent pathway may be a novel therapeutic target for treatment of ischemic retinopathy.

## Introduction

Ischemic retinopathy is one of the leading causes of visual impairment and irreversible blindness worldwide. It is a common clinical entity associated with various ocular disorders in which retinal blood flow is insufficient to meet the metabolic demands of the retina (the highest of any tissue), including acute glaucoma, retinal vein occlusion, retinopathy of prematurity, and diabetic retinopathy (Hardy et al., 2005; Jo et al., 2015). While it is well known that interruption of retinal blood flow causes retinal ischemia–reperfusion (RIR) injury and eventually leads to retinal ganglion cell (RGC) death (Fouda et al., 2018), molecular pathomechanisms of RGC death and associated treatment targets are still obscure. Microglia are the main immunological sentinels in the central nervous system (CNS), including retinal tissues. Under inflammatory conditions, microglia are activated and secret pro-inflammatory cytokines and cytotoxic mediators that can induce the death of neurons (Brown and Vilalta, 2015). Our previous studies demonstrated that this microglia-driven neuroinflammation mediates retinal tissue damage and RGC death during RIR injury (Chi et al., 2014).

In recent years, an increasing attention has been paid to the NOD-like receptors (NLRs) family which are the largest and most diverse cytoplasmic pattern recognition receptors in terms of the structure and function, as well as the signals they recognize (Meunier and Broz, 2017). Innate immune responses can be induced by the recognition of NLRs for endogenous danger signals, termed damage-associated molecular patters (DAMPs), or conservative microbial components, termed pathogen-associated molecular patterns (PAMPs) (Brubaker et al., 2015). According to their primary or best-characterized functions, NLRs can be categorized into three subgroups: inflammasome-forming NLRs, reproductive NLRs (functioning during reproduction and embryogenesis), and regulatory NLRs (Coutermarsh-Ott et al., 2016). While inflammasome-forming NLRs are well described, regulatory NLRs such as NOD1, NOD2, NLRX1, NLRC3, and NLRC5 are less studied and mainly refer to function as either positive or negative regulators of inflammatory signaling cascades (Coutermarsh-Ott et al., 2016; Fekete et al., 2018). In the past several years, accumulated evidence from *in vitro* and *in vivo* studies has clearly shown that inflammasome-forming NLRs, such as NLRP3, NLRP1, NLRP6, and NLRC4, play an important role in RIR injury (Chi et al., 2014; Wan et al., 2019; Chen et al., 2020). However, the contributions of regulatory NLRs to RIR injury are still unknown.

NLRC5 (NLR family, CARD domain containing 5) is a newly discovered NLRs family protein, belonging to the regulatory NLRs, which plays an important regulatory role in both adaptive and innate immune signaling (Meng et al., 2015; Benko et al., 2017). The role of NLRC5 in the regulation of major histocompatibility complex (MHC) class I genes is well established. However, the exact functions of NLRC5 in innate immune responses are unclear due to discrepancies and inconsistencies in reported data. Ma and Xie (2017) reported that NLRC5 deficiency aggravated fibrosis and inflammatory responses following heart injury, whereas Zhou et al. (2017) reported that NLRC5 silencing significantly ameliorated cardiac fibrosis. Others have reported that NLRC5 can act as a negative regulator of stress-associated nuclear factor kappa B (NF-κB) signaling, while other studies have found no influence of NLRC5 deficiency on the expression of NF-κB-dependent genes (Cui et al., 2010; Kumar et al., 2011; Yao et al., 2012). Moreover, the function of NLRC5 in the regulation of inflammasomes is even less well studied.

Programmed cell death (PCD), such as apoptosis, pyroptosis and necroptosis, is a fundamental cellular process essential for the homeostasis and development of multicellular organisms (Man et al., 2017). Our recent studies revealed that a NLRP12/NLRP3/NLRC4 inflammasome-initiated pyroptosis pathway contributes to RGCs death in elevated intraocular pressure (IOP)-induced retinal ischemia (Chen et al., 2020). Recent breakthroughs reveal that the different types of PCD are highly interconnected and could be regarded as a single, coordinated cell death system that allows for flexible mutual compensation, just like in some conditions inflammatory caspases can trigger apoptosis while apoptotic triggers can induce pyroptosis (Bedoui et al., 2020). However, the exact molecular mechanisms inducing RGC death by the microglial PCD, especially apoptosis and pyroptosis, are still not fully understood under ischemia or hypoxia conditions. Furthermore, whether and how NLRC5 regulates microglial PCD to mediate RIR injury is currently unknown.

In the present study, we elucidate novel regulatory functions of NLRC5 in promoting microglial pyroptosis and ensuing RGCs death under retinal ischemia. We further reveal the relationship between the canonical inflammasomes NLRP3/NLRC4 and NLRC5 in mediating microglial pyroptosis and apoptosis.

## Materials and Methods

### Ethics Statement

The adult female wild type C57BL/6 mice used in this study were purchased from GemPharmatech Co. Ltd., and raised in the Experimental Animal Center of Zhongshan Ophthalmic Center, Sun Yat-sen University, under specific pathogen-free conditions. All procedures involving animals were conducted strictly in accordance with the Association for Research in Vision and Ophthalmology (ARVO) Statement for the use of Animals in Ophthalmic and Vision Research. All animal experiments were formally reviewed and approved by the Animal Care and Ethics Committee of the Zhongshan Ophthalmic Center (Approval number: 2018-073). All efforts were made to ensure the welfare and alleviate the suffering of animals.

### Retinal Ischemic Injury Model

All animals were 6–8 weeks of age, 18–20 g, and in good health at the time of experiments. The RIR ischemic retinopathy model was established as described in our previous article (Chen et al., 2020). In brief, mice were injected intraperitoneally with 50 mg/kg pentobarbital sodium for general anesthesia and locally dropped with 0.5% tetracaine hydrochloride for corneal anesthesia. Pupils were dilated with 1% tropicamide, and then a 30-gauge needle (BD, Franklin Lakes, NJ, United States) linked to an elevated saline reservoir was inserted into the anterior chamber of the right eye to maintain IOP at 110 mmHg for 90 min. The contralateral eye served as a sham control. Retinal ischemia was confirmed by the disappearance of the fundus red-light reflex, conjunctival edema, and corneal haze, while subsequent reperfusion was confirmed by recovery of the red-light reflex. At different times during reperfusion, mice were sacrificed and eyeballs or retina tissues isolated for subsequent experiments.

A small interfering (si) RNA targeting NLRC5 (siNLRC5) was used to silence NLRC5 expression in mouse retina. To avoid degradation *in vivo*, the siRNA was specifically designed and modified by methylation and cholesterol. A 1-nM solution of either siNLRC5 or a negative control siRNA (siNC) was injected into the vitreous cavity before the onset of reperfusion. The negative control siRNA was used to illustrate the specificity of siNLRC5 action and serve as a reference to analyze the siNLRC5 action. Both siRNAs were purchased from Ribobio Co., Ltd. The target sequence for the NLRC5-siRNA was TGACCAGCAGACTCTTTGA. The mice were sacrificed 3 days after intravitreal siRNA injection.

### Histology and Immunohistochemistry

At designated times post-reperfusion, mice were sacrificed and their eyeballs isolated for histology and immunohistochemistry assays. The enucleated eyes were fixed with 4% paraformaldehyde overnight and then embedded in paraffin. Three, 4-μm thick slices through the optic disk of each eye were selected for hematoxylin and eosin (HE) staining. To quantitatively assess retinal tissue damage, total retinal thickness between the inner and outer limiting membranes was measured at a distance of 1 mm from the optic disk using CaseViewer software (3DHISTECH). Measurements were averaged to yield a representative retinal thickness for each eye. Slices selected for immunohistochemical analysis were deparaffinized and treated with antigen retrieval. According to the manufacturer’s instructions, TUNEL assay was performed to detect and quantify the PCD at single cell level by using *In Situ* Cell Death Detection Kit (Roche). TUNEL-positive cells were manually counted in inner retina and the number of TUNEL-positive cells was expressed as the average per 1-mm length area using Image Pro Plus 7.0 as previously described (Sun et al., 2010; Sato et al., 2020). For TUNEL assay on cultured cells, the average TUNEL positive cell number in the percentage of total DAPI-stained nuclei was calculated. For immunofluorescence staining of the retina, the sections after antigen retrieval were first permeabilized with 0.3% Triton X-100 at room temperature for 30 min, blocked with 10% goat serum at room temperature for 1 h, and then incubated at 4°C overnight with primary antibodies targeting Rabbit polyclonal anti-RBPMS (1:200, Abclonal, Cat#ab152101), Rabbit polyclonal anti-Iba-1 (1:200, Wako, Cat#019-19741), Goat polyclonal anti-Iba-1 (1:100, abcam, Cat#ab48004), Mouse monoclonal anti-GSDMD (1:200, Santa Cruz, Cat#sc-393581), and Mouse monoclonal anti-NLRC5 (1:100, Santa Cruz, Cat#sc-515668). Sections were incubated with appropriate secondary antibodies at room temperature under darkness for 1 h and then cell nuclei were counterstained with DAPI. Fluorescence images were acquired using an Olympus fluorescence microscope or a Nikon A1 spectral confocal microscope. RBPMS is a specific and reliable marker for RGCs (Kwong et al., 2010). To perform RGC survival counts, each RBPMS-positive cell was merged with DAPI and counted individually to represent number of RGCs. RGC survival counts of each group were determined by counting average RBPMS-positive cells from four retina of four different animals (per retina including three 4-μm thick slices from ora serrata to ora serrata through the optic disk). The average of the RBPMS-positive cells counts in the NC siRNA normal control group as the denominator to calculate the percentages in the remaining groups. To detect the expression of NLRC5 protein, immunohistochemical staining was performed using a DAB kit (Servicebio) following the standard protocol. Briefly, sections were deparaffinized, subjected to antigen retrieval, incubated in 3% hydrogen peroxide to eliminate endogenous peroxidase activity, blocked in solution containing 3% bovine serum albumin (BSA) and 0.3% Triton X-100, and incubated with primary antibody targeting NLRC5 (1:100, abcam, Cat#ab105411). Images were acquired using an Olympus light microscope.

### Cell Culture and Treatment

The BV2 microglial cell line was purchased from Procell Co. Ltd. (#CL-0493, Wuhan, China) and cultured in a humidified atmosphere of 5% CO_2_ at 37°C. The growth medium consisted of Dulbecco’s Modified Eagle’s Medium (DMEM) supplemented with 10% fetal bovine serum (FBS) and 1% penicillin–streptomycin. For gene silencing, BV2 cells were transfected with NLRC5-targeted or control siRNA when the cell density reached 60%–80% confluence using Lipofectamine RNAiMAX (Invitrogen) according to the manufacturer’s instructions. Briefly, the Lipofectamine RNAiMAX reagent and siRNA were both diluted in Opti-MEM(Gibco) medium, then the diluted siRNA and the diluted Lipofectamine RNAiMAX solution were mixed in a 1:1 ratio and incubated for 5 min and finally the siRNA-lipid complex was added to the cells. After incubation for 24 h, the cells were treated with oxygen–glucose deprivation and reperfusion.

### *In vitro* Oxygen–Glucose Deprivation and Reperfusion (OGDR) Model

The OGDR model was used to simulate the effects of retinal ischemia on microglia in isolation. Cultures of BV2 cells were first starved with a serum-free and glucose-free DMEM for 1 h under normoxic conditions, and then exposed to hypoxia (95% N_2_, 5% CO_2_) for 3 h in an incubator chamber. Alternatively, control cultures were incubated in serum-free DMEM under normoxic conditions for the same duration. Reperfusion was started by replacing medium with DMEM containing 10% FBS and 4.5 g/L glucose, and culturing them at 37°C for 24 h under normoxic conditions.

### Western Blotting

Total protein was extracted using M-PER mammalian protein lysis buffer (Thermo Fisher Scientific, Rockford, IL, United States) supplemented with protease inhibitor cocktail (Thermo Fisher Scientific, Rockford, IL, United States) (1:100) according to the manufacturer’s instructions. The protein concentration was determined using a quantitative BCA protein kit (Generay, Shanghai, China). Proteins were then separated using 8% or 10% polyacrylamide gels and transferred onto polyvinylidene difluoride (PVDF) membranes following standard protocols. Membrane were blocked with 5% non-fat milk diluted in Tris-buffered saline plus Triton-X (TBST) for 1 h at room temperature, and then incubated at 4°C overnight with the following primary antibodies: Rabbit polyclonal anti-NLRP3 (1:500, ABclonal, Cat#A14223), Rabbit polyclonal anti-NLRC5 (1:250, abcam, Cat#ab105411), Rabbit monoclonal anti-caspase-3 (1:200, Cell Signaling Technology, Cat#9665), Rabbit polyclonal anti-cleaved caspase-3 (1:200, Cell Signaling Technology, Cat#9661), Rabbit polyclonal anti-caspase-8 (1:200, ABclonal, Cat#A11324), Rabbit monoclonal anti-cleaved caspase-8 (1:200, Cell Signaling Technology, Cat#8592), Rabbit polyclonal anti-caspase-1 (1:200, ABclonal, Cat#A0964), Mouse monoclonal anti-caspase-1 p20 (1:200, AdipoGen, Cat#AG-20B-0042), Rabbit monoclonal anti-GSDMD (1:200, abcam, Cat#ab209845), Rabbit polyclonal anti-IL-1β (1:200, Immunoway, Cat#YT5201), Rabbit polyclonal anti-NLRC4 (1:500, ABclonal, Cat#A7382), and Rabbit monoclonal anti-β-actin (1:80000, ABclonal, Cat#AC026) as the gel loading control. Finally, after incubation in corresponding secondary antibodies, the immunoblots were detected by an enhanced chemiluminescence kit (ECL, eBioscience), captured using an Image Lab system (Bio-Rad, United States), and quantified using ImageJ software.

### Immunoprecipitation

After the protein supernatant was extracted, immunoprecipitation antibody targeting Mouse monoclonal anti-NLRC5 (1:100, Santa Cruz, Cat#sc-515668) or negative control mouse IgG (1:100, ABclonal, Cat#AC011) was added and incubated overnight at 4°C. Next, 30 mL Protein A/G-agarose beads (Abmart, A10001) were added and incubated for 3 h. After incubation, the agarose beads were separated by instantaneous centrifugation and washed three times with cell lysis buffer. Then, beads were added with 60 mL 2 × loading buffer and boiled for 5 min, and removed after centrifugation. The eluted immune complexes in the supernatant were analyzed by western blotting. Cell lysates directly used for western blot analysis without immunoprecipitation processing were used as positive controls.

### Quantitative Real-Time PCR (RT-qPCR)

Total RNA was extracted from cells or retinal tissue samples using TRIzol reagent (Thermo Fisher Scientific) according to the instruction manual and reverse-transcribed into cDNA using HiScript^®^ III RT SuperMix (Vazyme). Real-time quantitative PCR (RT-qPCR) was conducted using ChamQ^TM^ SYBR^®^ Color qPCR Master Mix (Vazyme) according to the manufacturer’s standard procedure and recorded by a Light Cycler 480 Real-Time PCR system. The sequences of primers were listed in Table 1.

**TABLE 1:** Primer sequences used for this study.

**Targeted**	**Forward primer sequence**	**Reverse primer sequence**
**genes**	**(5′–3′)**	**(5′–3′)**
mNLRC4	ACCTGGAAAAGGATGGGAA TGAA	AAGTTTGGCAAGTCCTGGGG
mGSDMD	GCGATCTCATTCCGGTGGA CAG	TTCCCATCGACGACATCAGA GAC
mNLRP3	ATTACCCGCCCGAGAAAGG	TCGCAGCAAAGATCCACACAG
mNLRC5	TCAGCCCAGAACAAGTATCC	TGGGCACAGACTTCCATTAG
mIL-1β	CTCCATGAGCTTTGTACAAGG	TGCTGATGTACCAGTTGGGG
mCaspase-1	CAGGCAAGCCAAATCTTTATC ACT	GTGCCATCTTCTTTGTTCTGT TCTT
mCaspase-8	GTCACCGTGGGATAGGATACA	AGACATAACCCAACTCCGAAAA
mCaspase-3	TGGTGATGAAGGGGTCATTTA TG	TTCGGCTTTCCAGTCAGACTC
mβ-Actin	TCCAGCCTTCCTTCTTGGGT	GCACTGTGTTGGCATAGAGGTC

### Statistical Analysis

All statistical analyses were conducted using GraphPad Prism 6.0 software (GraphPad Software, Inc., San Diego, CA, United States). After checking the data for the normal distribution by Shapiro-Wilk test using SPSS 25.0 (IBM SPSS, Inc., Chicago, IL, United States), data are expressed as mean ± SD. Two group means were compared using the two-tailed independent samples Student’s *t*-test while three or more group means were compared using one-way or two-way ANOVA with *post hoc* Bonferroni tests. A *P* < 0.05 was considered statistically significant for all tests.

## Results

### NLRC5 Expression Is Markedly Elevated During RIR Injury

**FIGURE 1:**
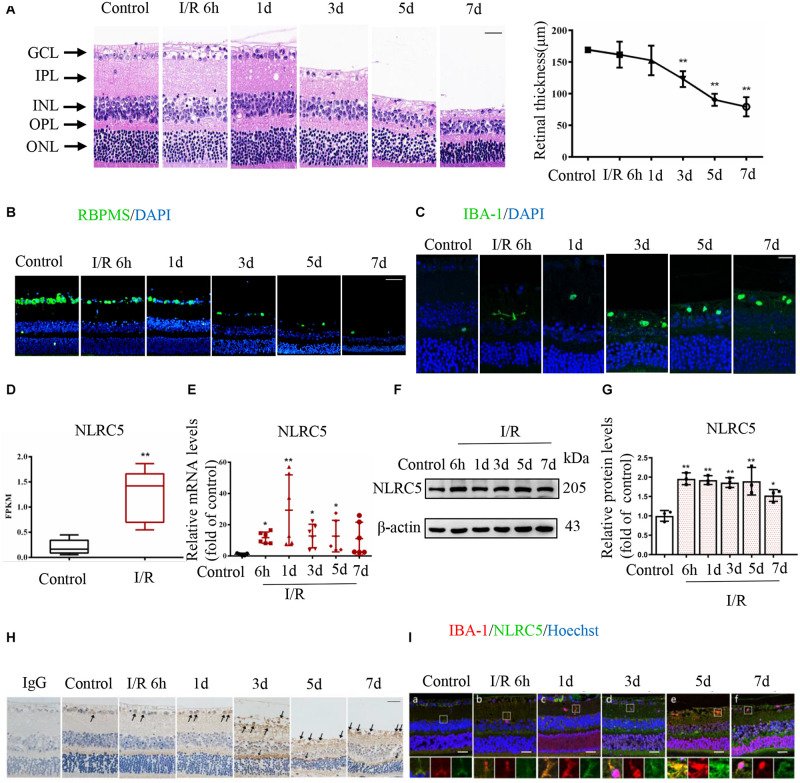
The regulatory NOD-like receptor NLRC5 is upregulated during elevated intraocular pressure (IOP)-induced retinal ischemia–reperfusion (RIR) injury. **(A)** Hematoxylin and eosin (HE) staining of retinal sections showing the evolution of tissue and cellular damage during reperfusion and corresponding quantitative analysis demonstrating progressively reduced retinal thickness between the inner and outer limiting membranes (Scale bar: 30 μm, Magnification: ×400, *n* = 5). **(B,C)** Immunofluorescence images showing reduced numbers of anti-RBPMS-labeled retinal ganglion cells (RGCs) (Scale bar: 50 μm, Magnification: ×200) and elevated numbers of anti-Iba-1-labeled microglia (Scale bar: 15 μm, Magnification: ×400) during RIR. **(D)** Box plot illustrating significantly increased NLRC5 mRNA expression in the retina of RIR injury based on RNA sequencing data from our recently published article (*n* = 5). **(E–G)** RT-qPCR and western blot analysis showing elevated NLRC5 expression following RIR at mRNA (*n* = 6) and protein levels (*n* = 3), respectively. **(H)** Immunohistochemical analysis showing elevated NLRC5 expression during RIR (Scale bar: 30 μm, Magnification: ×400, *n* = 3). **(I)** Dual immunofluorescence image indicating anti-Iba-1-labeled microglia (red) and NLRC5 (green) (Scale bar: 25 μm, Magnification: ×400). I/R, retinal ischemia–reperfusion; ONL, outer nuclear layer; OPL, outer plexiform layer; INL, inner nuclear layer; IPL, inner plexiform layer; GCL, ganglion cell layer. Results **(A,D,E,G)** are presented as mean ± SD. *P < 0.05, **P < 0.01. Two-tailed unpaired Student’s *t*-test and one-way ANOVA with *post hoc* Bonferroni tests was applied.

First, we established a high IOP-induced RIR injury model simulating the pathological process of ischemic retinopathy. Model mice demonstrated the typical morphological changes of retinal tissue damage during reperfusion as well as a significant reduction in retinal thickness, especially of the inner retina, from the third day of reperfusion and continuing thereafter (Figure 1A). Additionally, immunofluorescence staining revealed a gradual decrease in the number of RGCs and a concomitant increase in the number of activated microglia as evidenced by the transition from a ramified to amoeboid morphology (Figures 1B,C).

Mounting evidence implicates inflammasome-forming NLRs such as NLRP3, NLRP1, and NLRP6 in RIR injury (Chi et al., 2014; Wan et al., 2019), but the potential roles of regulatory NLRs have not been explored. By analyzing transcriptome sequencing data obtained from our recently published study (Chen et al., 2020), we discovered that the mRNA expression level of regulatory NLRC5 was significantly upregulated in the retina of RIR injury model mice, and this upregulation was further confirmed by RT-qPCR (Figures 1D,E). Both western blot and immunohistochemical staining also demonstrated significantly upregulated expression of NLRC5 in the retina at different times during reperfusion (Figures 1F–H). Moreover, dual immunofluorescence staining further revealed upregulated NLRC5 expression in retinal microglia (Figure 1I).

### NLRC5 Deficiency Ameliorates Retinal Damage and RGC Death During RIR Injury

**FIGURE 2:**
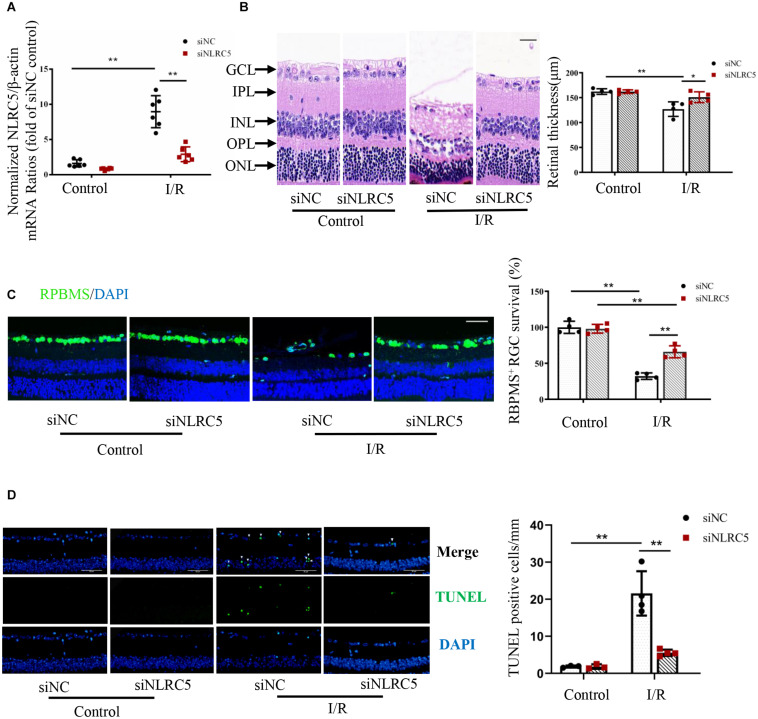
NLRC5 knockdown effectively ameliorates retinal damage following ischemia–reperfusion. **(A)** RT-qPCR analysis of retinal NLRC5 gene knockdown efficiency using a targeted siRNA (*n* = 6). **(B)** HE staining of mouse retina showing that intravitreal injection of NLRC5 siRNA ameliorates tissue damage and reverses the reduction in retinal thickness following ischemia–reperfusion compared to retinas transfected with control siRNA (Scale bar: 30 μm, Magnification: ×400, *n* = 4). **(C)** Retinal immunofluorescence images and corresponding quantitative analysis of anti-RBPMS-labeled RGCs showing that the reduction in RGC number following RIR is reversed by NLRC5 knockdown (Scale bar: 50 μm, Magnification: ×200, *n* = 4). **(D)** TUNEL staining of retina following RIR (Scale bar: 50 μm, Magnification: ×400, *n* = 4). The white arrow indicates TUNEL-positive cells. All samples were obtained on the third day of reperfusion. I/R, retinal ischemia–reperfusion; ONL, outer nuclear layer; OPL, outer plexiform layer; INL, inner nuclear layer; IPL, inner plexiform layer; GCL, ganglion cell layer. Results are presented as mean ± SD. *P < 0.05, **P < 0.01. Two-way ANOVA with *post hoc* Bonferroni tests was applied.

To examine the functions of NLRC5 in RIR injury, we compared retinal responses to high IOP between mice receiving intravitreal injection of a siRNA targeting the NLRC5 gene and mice receiving control siRNA. First, NLRC5 gene silencing was confirmed by RT-qPCR analysis, which showed that the siRNA targeting NLRC5 significantly down-regulated the expression of NLRC5 in RIR injury compared to the control siRNA (Figure 2A). HE staining revealed that NLRC5 knockdown notably reduced the severity of retinal tissue damage and the decrease in total retinal thickness observed during reperfusion, compared with the control siRNA (Figure 2B). Further, immunofluorescence staining also showed greater numbers of surviving RGCs (Figure 2C) and reduced numbers of TUNEL-positive cells (Figure 2D) in the retinal ganglion cell layer (GCL) and inner nuclear layer (INL) of the NLRC5 knockdown group compared to the control siRNA group.

### Pyroptosis and Apoptosis Are Essential to the Development of Retinal Ischemic Injury

**FIGURE 3:**
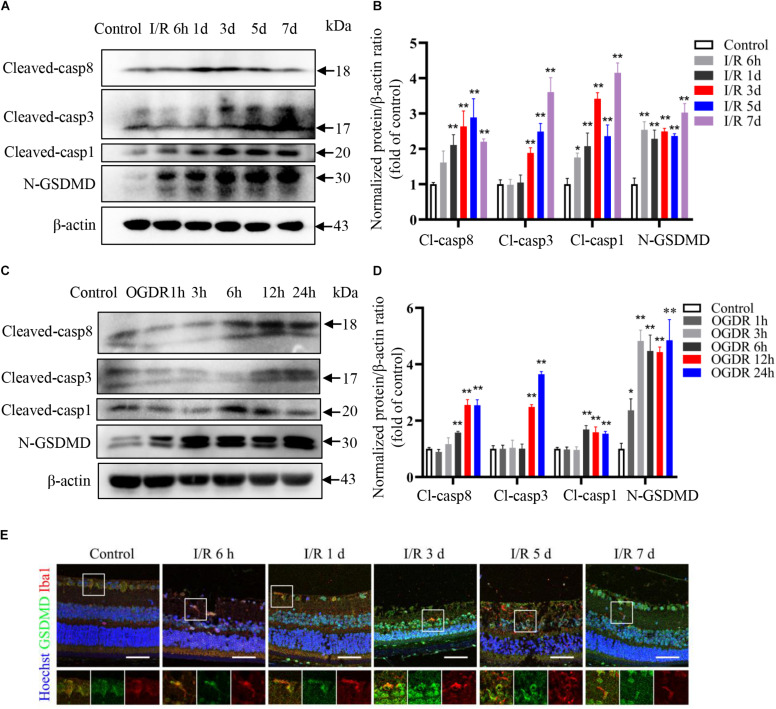
Pyroptosis and apoptosis are induced sequentially by ischemia–reperfusion in retina and cultured microglia. **(A,B)** Western blot analysis showing induction of pyroptosis and apoptosis markers in retinal tissue following RIR (*n* = 3). **(C,D)** Western blot analysis showing upregulation of pyroptosis and apoptosis markers in BV2 microglia following OGDR (*n* = 3). **(E)** Dual immunofluorescence staining of retina showing induction of the pyroptosis markers GSDMD in retinal microglia following RIR (Scale bar: 50 μm, Magnification: ×400). I/R, retinal ischemia–reperfusion; OGDR, oxygen-glucose deprivation and reperfusion. *P < 0.05, **P < 0.01. One-way ANOVA with *post hoc* Bonferroni tests was applied.

Multiple processes, including neuroinflammation, oxygen free radical generation, intracellular calcium overload, and activation of apoptosis signaling pathways contribute to the development of RIR injury (Takahashi et al., 1992; Kuriyama et al., 2001; Oz et al., 2005; Madeira et al., 2016). Our recent study also indicated a key role for pyroptosis in elevated IOP-induced retinal ischemic injury (Chen et al., 2020). Consistent with these findings, protein levels of the apoptosis markers cleaved caspase-8 and cleaved caspase-3 were significantly upregulated starting on day 3 after reperfusion *in vivo*, while the protein levels of pyroptosis markers cleaved caspase-1 and N-terminal GSDMD increased significantly as early as 6 h following reperfusion (Figures 3A,B).

To examine pyroptotic and apoptotic signaling specifically in microglia, we established an *in vitro* OGDR model using BV2 microglia and found that the protein expression levels of pyroptosis markers increased more rapidly than apoptosis markers during reperfusion (Figures 3C,D), consistent with *in vivo* results. Dual immunofluorescence staining further confirmed increased expression level of pyroptosis marker GSDMD in retinal microglia during reperfusion (Figure 3E).

### NLRC5 Is an Essential Mediator of Microglial Pyroptosis

Microglia, the primary resident immune cell in the retina, make pathologic contributions to augmenting RGC degeneration by producing neurotoxic proinflammatory cytokines and by clearing stressed but still living neurons (Silverman and Wong, 2018). However, the contributions of NLRC5 signaling to the proinflammatory activity of microglia as well as to the observed microglial pyroptosis and apoptosis during RIR injury have not been examined.

**FIGURE 4:**
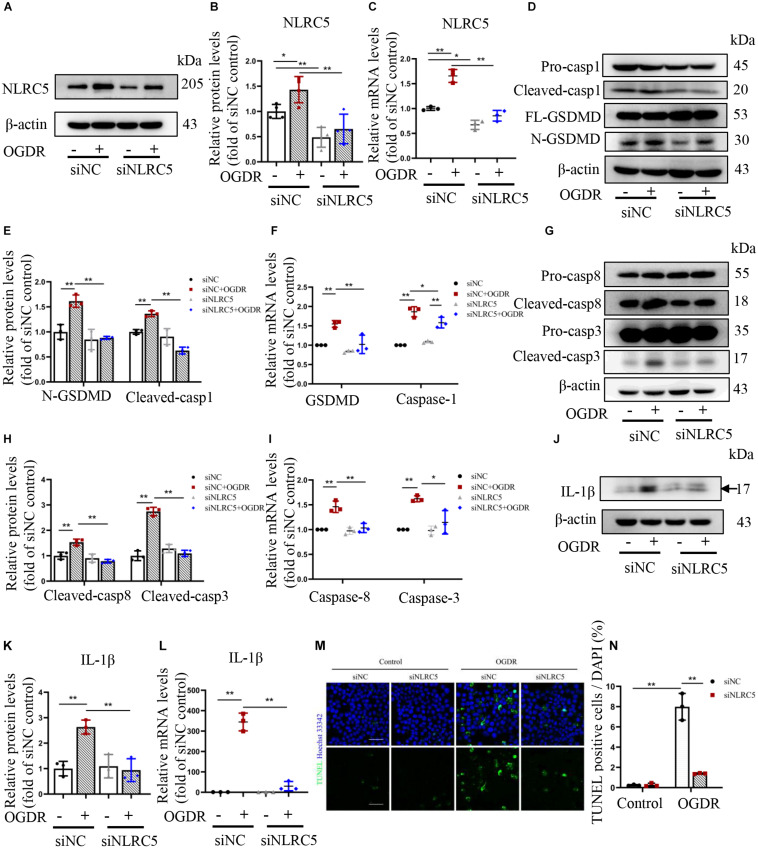
Knockdown of NLRC5 inhibits OGDR-induced BV2 cells apoptosis, pyroptosis, and IL-1β production. **(A–C)** Western blot and RT-qPCR analysis of NLRC5 expression to verify gene knockdown efficiency. **(D–L)** Western blot and RT-qPCR analysis showing that NLRC5 knockdown reverses the OGDR-induced upregulation of pyroptosis markers **(D–F)**, apoptosis markers **(G–I)** and mature IL-1β **(J–L)**. **(M,N)** TUNEL staining of BV2 cells following OGDR (Scale bar: 50 μm, Magnification: ×400). All samples were obtained after 24 h of reperfusion. All data shown are representative of at least three independent experiments and presented as mean ± SD. *P < 0.05, **P < 0.01. Two-way ANOVA with *post hoc* Bonferroni tests was applied. I/R, retinal ischemia–reperfusion; OGDR, oxygen-glucose deprivation and reperfusion.

Therefore, we explored the influence of NLRC5 knockdown on pyroptosis and apoptosis of BV2 microglia subjected to *in vitro* OGDR. Transfection with a siRNA targeting NLRC5 significantly inhibited its expression at both protein and mRNA levels regardless of OGDR exposure, compared with the control siRNA (Figures 4A–C). In the control siRNA groups, OGDR induced pyroptosis and apoptosis of BV2 microglia, as demonstrated by the increased expression of cleaved caspase-1, N-terminal GSDMD, cleaved caspase-3 and cleaved caspase-8, as well as increased numbers of TUNEL-positive cells (Figure 4). Silencing NLRC5 significantly reduced the elevation in cleaved caspase-1 and N-terminal GSDMD induced by OGDR at both the protein level and mRNA level (Figures 4D–F). Moreover, NLRC5 knockdown also significantly reduced OGDR-induced elevations in cleaved caspase-3 and -8 at both the protein level and mRNA level (Figures 4G–I). Furthermore, NLRC5 knockdown inhibited OGDR-enhanced production of mature IL-1β (Figures 4J–L) as well as OGDR-increased numbers of TUNEL-positive cells (Figures 4M,N) compared with the control siRNA.

### NLRC5 Directly Binds and Promotes NLRP3/NLRC4 Inflammasomes to Mediate Pyroptosis

**FIGURE 5:**
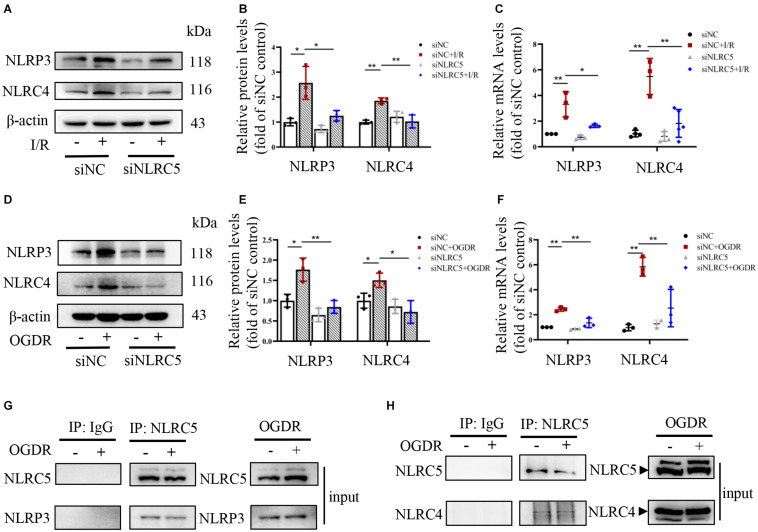
NLRC5 binds to and activates NLRP3/NLRC4 inflammasomes during RIR or OGDR. **(A–F)** Western blot and RT-qPCR analysis of NLRP3 and NLRC4 expression in BV2 microglial cultures and mouse retina following OGDR and RIR, respectively. Expression levels of both proteins and mRNAs were elevated *in vivo* and *in vitro* by RIR or OGDR. Elevated expression levels following OGDR were markedly suppressed by NLRC5 knockdown. **(G,H)** Co-immunoprecipitation assay showing that NLRC5 interacts with NLRP3/NLRC4 in BV2 cells subjected to OGDR. All data shown are representative of at least three independent experiments and presented as mean ± SD. *P < 0.05, **P < 0.01. Two-way ANOVA with *post hoc* Bonferroni tests was applied. I/R, retinal ischemia–reperfusion; OGDR, oxygen-glucose deprivation and reperfusion.

In response to PAMPs or DAMPs, canonical inflammasomes containing NLRP3 or NLRC4 recruit and activate pro-caspase-1, which then can cleave GSDMD to initiate pyroptosis, as well the precursors of proinflammatory IL-18 and IL-1β (Aglietti and Dueber, 2017). Recent work has also revealed that inflammasomes can recruit pro-caspase-8, thereby triggering apoptosis (Aachoui et al., 2013). However, it is unknown if NLRC5 can also induce pyroptosis and apoptosis by regulating canonical inflammasomes during RIR injury. Protein and mRNA expression levels of NLRP3 and NLRC4 increased significantly in the control siRNA groups under RIR *in vivo* and OGDR *in vitro* (Figures 5A–F). While these elevations induced by OGDR or RIR were significantly suppressed by NLRC5 knockdown (Figures 5A–F). Immunoprecipitation analysis further showed that NLRC5 can bind directly to NLRP3 and NLRC4 in BV2 microglia (Figures 5G,H), suggesting that NLRC5 may form a complex with NLRP3/NLRC4 and positively regulate NLRP3/NLRC4 inflammasome pathways.

## Discussion

Ischemia–reperfusion injury is the major pathogenic process underlying multiple forms of ischemic retinopathy, and often causes irreversible visual impairment or even blindness, severely damaging the patient’s quality of life (Osborne et al., 2004). The retina is particularly sensitive to ischemia and hypoxia due to its high metabolic requirements. The process of RIR injury induces oxidative stress and a series of inflammatory cascades, which in turn can destroy retinal neurons, especially RGCs. Unfortunately, once dead, RGCs can hardly regenerate, just like other neurons in the CNS, and eventually causes irreversible loss of vision (Liu et al., 2019). Therefore, a better understanding of the molecular mechanisms leading to ischemia-induced RGC dysfunction and death is essential for the development of therapeutic strategies to delay or halt vision loss. In the current study, we provide the first evidence that the regulatory NOD-like receptor NLRC5 is critically involved in elevated IOP-induced retinal ischemic injury and RGC death. We also uncovered a crucial function for NLRC5 in mediating neuroinflammatory responses and regulating both apoptosis and pyroptosis pathways in microglia. Furthermore, our findings for the first time reveal the connection between NLRC5 and NLRC4, and indicate that NLRC5 contributes to RIR injury mainly by directly binding to NLRP3/NLRC4 inflammasomes and promoting their activation.

High IOP-induced RIR injury model is a widely used and well-established model to simulate the pathological process of ischemic retinopathy (Hartsock et al., 2016). Successful modeling is commonly evaluated by whether neuron loss in the GCL and retinal tissue thinning are significant compared to normal controls, although the degree of ischemic damage varies due to differences in modeling methods in different studies, such as the difference in the degree of elevated IOP (Wang et al., 2016; Huang et al., 2018; Wan et al., 2019). In this study, IOP was elevated to 110 mmHg for 90 min and elevated IOP-induced ischemic damage was also confirmed by significant retinal tissue thinning and RGCs loss from the third day of reperfusion. The diverse functions of inflammasome-forming NLRs family members are well characterized, while the potential roles of regulatory NLRs are less extensively explored. We first discovered that regulatory NLRC5 expression was markedly upregulated in the murine retina under RIR injury, implying its potential association with ischemic damage. Evidence shows that microglial responses are associated with the severity of RGCs degeneration by producing pro-inflammatory and neurotoxic factors (Almasieh et al., 2012; Silverman and Wong, 2018). We found that NLRC5 was also upregulated in retinal microglia during ischemia, suggesting that NLRC5 may exert its function though retinal microglia. Indeed, knockdown of NLRC5 expression in cultured microglia reduced cytotoxic IL-1b production in response to *in vitro* ischemia, further supporting this notion.

NLRC5 is the largest and most recently discovered member of the NLRs family, and has recently been implicated in the development of inflammatory diseases (Wang et al., 2019); however, precise functions in innate immune responses remain controversial. Ma and Xie (2017) reported that NLRC5 deficiency aggravated fibrosis and inflammatory responses triggered by high fat-induced heart injury). Conversely, Zhou et al. (2017) reported that NLRC5 silencing significantly inhibited cardiac fibroblast proliferation and migration, and ameliorated TGF-β1-induced fibrosis. Li et al. (2020) reported that NLRC5 partially aggravated inflammatory lung injury, while Wang et al. (2020) concluded that NLRC5 negatively regulated this inflammatory response. The contributions of NLRC5 to ophthalmopathy are similarly unclear. We therefore explored the potential role of NLRC5 *in vivo* and *in vitro* models that simulate the pathological process of ischemic retinopathy. Our findings provide the first evidence that NLRC5 deficiency can ameliorate retinal damage and RGCs death in mice subjected to high IOP-induced retinal ischemic injury, suggesting that NLRC5 is an important contributor to the pathophysiology of ischemic retinopathy and a promising therapeutic target.

An electron microscopy study of degenerating retinal cells in the GCL and INL at several time points after RIR injury by Büchi et al. revealed three morphologically distinct types of cell death, one consistent with necrosis, one resembling apoptosis, and one sharing several features with necrosis (Büchi, 1992). Also, accumulating evidence suggests that inhibition of the apoptosis pathway attenuates retinal ischemic injury (Ishikawa et al., 2012; Gencer et al., 2014; Han et al., 2020). In the current study as well, we confirmed activation of the apoptosis pathway in high IOP-induced retinal ischemic injury. Recently, a newly identified form of pro-inflammatory PCD called pyroptosis has also been implicated in various neurodegenerative diseases. Pyroptosis involves activation of caspase-1 or caspase-11/4/5, which then cleaves GSDMD to form N-terminal GSDMD. These cleaved N-terminal GSDMD proteins accumulate in the plasma membrane and form pores, eventually leading to plasma membrane rupture and release of cytosolic contents (Kovacs and Miao, 2017). This study, together with our recently published study, provide compelling evidence that pyroptosis of activated microglia contributes to retinal ischemic injury, including the death of RGCs (Chen et al., 2020). We also found that both apoptosis and pyroptosis pathways were activated in OGDR-stimulated microglia, and that the expression of pyroptotic marker proteins increased more rapidly than apoptotic marker proteins. Poh et al. (2018) also found apoptotic and pyroptotic cell death of microglia under cerebral ischemia. These distinct PCD pathways are interconnected functionally and at the molecular levels, and thus may not operate in isolation but rather act cooperatively or synergistically for mutual compensation (Bedoui et al., 2020). Bedoui et al. (2020) also provide an explanation for the phenomenon that the onset of apoptosis is delayed than that of pyroptosis, that is, they believe that it is because the onset of apoptosis involves more steps to ensure coordinated operation. Clearly, more work will be needed to understand the precise molecular and physiological interrelationships between pyroptosis and apoptosis pathways and how they work together to induce RGCs death.

Previous studies have shown that NLRC5 can mediate apoptosis in acute kidney injury, cerebral ischemia/reperfusion injury, and acute myocardial infarction (Han et al., 2018; Liu et al., 2020; Yang et al., 2020). However, little is known about the function of NLRC5 in the apoptosis pathway during RIR injury. We found that NLRC5 knockdown reduced production of the key apoptotic effector cleaved caspase-3, suggesting that NLRC5 can promote microglial apoptosis. Moreover, to our knowledge, no reports have shown whether NLRC5 plays a role in the pyroptosis pathway. We show for the first time that NLRC5 knockdown can also reduce production of both the key pyroptosis effector protein N-terminal GSDMD and bioactive IL-1β, suggesting that NLRC5 can promote microglial pyroptosis and the production and release of neurotoxic inflammatory mediators.

Inflammasomes are intracellular multiprotein signaling complexes that are classically composed of three parts: a cytosolic sensor such as nucleotide-binding domain and leucine-rich repeat receptor (NLR) or absent in melanoma 2-like receptor (ALR), an adaptor protein such as apoptosis-associated speck-like protein containing a CARD (ASC), and an effector caspase precursor such as pro-caspase-1 (Rathinam and Fitzgerald, 2016). Recent work indicates that inflammasomes can trigger pyroptotic and apoptotic cell death in response to DAMPs or PAMPs by recruiting and activating effector caspases including caspase-1 and/or caspase-8 (Aachoui et al., 2013; Sagulenko et al., 2013). In view of the novel function of NLRC5 in microglial pyroptosis and apoptosis, we therefore hypothesize that a regulatory link between NLRC5 and inflammasomes is essential for the development of RIR injury. Triantafilou et al. (2013) used fluorescence resonance energy transfer to reveal that NLRC5 synergistically promotes the assembly of NLRP3 inflammasome in response to rhinovirus infection, and Davis et al. (2011) used co-immunoprecipitation to demonstrate that NLRC5 directly binds with NLRP3 and ASC in a nucleotide-binding domain-dependent manner. However, the association between NLRC5 and other inflammasomes such as NLRC4 inflammasome is less studied. Our findings not only confirm that NLRC5 can bind with NLRP3 to mediate NLRP3 inflammasome activation but also reveal that NLRC5 directly acts with NLRC4 to synergistically promote NLRC4 inflammasome activation, suggesting that the regulation of pyroptosis and apoptosis by NLRC5 depends primarily on its direct combination with NLRP3/NLRC4 to promote the activation of inflammasomes, co-inducing RGCs death.

In conclusion, this study demonstrates for the first time that the regulatory NLR NLRC5 can mediate retinal tissue injury and RGCs death during RIR injury. We also demonstrate that NLRC5 knockdown can reduce neuroinflammation and inhibit pyroptosis and apoptosis pathways in activated microglia under ischemia or hypoxia conditions by binding to NLRP3/NLRC4 and preventing the activation of inflammasomes and ensuing pro-inflammatory cytokine production. Our findings suggest that NLRC5 has an essential role in the regulation of high IOP-induced retinal ischemic injury and microglial pyroptosis, highlighting NLRC5 as a promising therapeutic target to treat ischemic retinopathy.

## Data Availability Statement

The original contributions presented in the study are included in the article/supplementary material, further inquiries can be directed to the corresponding author.

## Ethics Statement

The animal study was reviewed and approved by the Animal Care and Ethics Committee of the Zhongshan Ophthalmic Center (Approval number: 2018-073).

## Author Contributions

WC and YD contributed to the study concept and design. WC, YD, LS, LoS, JL, and YF contributed to the experimental and technical support. WC, YD, LiS, CY, and YF contributed to the acquisition, analysis, or interpretation of data. WC, YD, and LiS contributed to the RNA sequence analysis. WC and YD contributed to the drafting of the manuscript. WC, YD, and LoS contributed to the critical revision of the manuscript for important intellectual content. WC, YD, and YF contributed to the statistical analysis. All authors contributed to the article and approved the submitted version.

## Conflict of Interest

The authors declare that the research was conducted in the absence of any commercial or financial relationships that could be construed as a potential conflict of interest.
